# Losartan-induced Pancreatitis

**DOI:** 10.7759/cureus.6253

**Published:** 2019-11-28

**Authors:** Shamsuddin M Anwar, Anum Aqsa, Rameez Shaukat

**Affiliations:** 1 Internal Medicine, Staten Island University Hospital, Staten Island, USA

**Keywords:** pancreatitis, ace inhibitors, angiotensin receptor blockade

## Abstract

Drug-induced acute pancreatitis (DIAP) is a rare gastrointestinal condition but well-known in the medical literature. The medications have been classified into four subgroups (Classes I-IV) depending upon the propensity of the cases discussed in the literature, interval time period between drug initiation to pancreatitis, and reaction to the drug with reintroduction. Our clinical case is one such example where losartan was described as the agent of recurrent pancreatitis after excluding all other possible causes with laboratory and imaging studies.

## Introduction

Acute pancreatitis is a frequently seen gastrointestinal condition in clinical settings, and its common causes include gallstones, hypertriglyceridemia, excess alcohol usage, iatrogenic causes, such as endoscopic retrograde cholangiopancreatography (ERCP), hypercalcemia, and drugs such as certain antiretrovirals, immunosuppressants, diuretics, and antibiotics. We intend to describe one such case of drug-induced acute pancreatitis (DIAP) with renin-angiotensinogen system inhibitor-losartan.

## Case presentation

A 71-year-old female with a pertinent history of essential hypertension, anxiety, and hypothyroidism presented with acute-onset abdominal pain. The pain described the typical characteristics of pancreatitis, with sharp mid-gastric pain radiating towards the back, without any significant relieving factors, accompanied by unremitting nausea and vomiting. The patient did not have any recent history of smoking, drinking, or any recreational drug use. Her home medications included losartan, Synthroid, Xanax, and bupropion.

Clinical history did not yield any relevant information, apart from the fact that the patient had presented with similar acute symptoms of pancreatitis approximately four weeks prior to this episode. She was conservatively managed with intravenous fluids and pain control with the resolution of symptoms. The patient was investigated for common causes of pancreatitis, including ultrasound and computed tomography (CT) of the abdomen, lipid profile, drug screen, and hepatitis panel. The workup to rule out the common causes of pancreatitis was negative.

The patient subsequently improved and was discharged home to be followed by the gastroenterology for further investigations, including magnetic resonance imaging (MRI) of the abdomen and immunoglobulin (IgG4) levels, to rule out uncommon causes of pancreatitis. Of note, before discharge home, she was resumed on losartan at the same dose.

**Figure 1:**
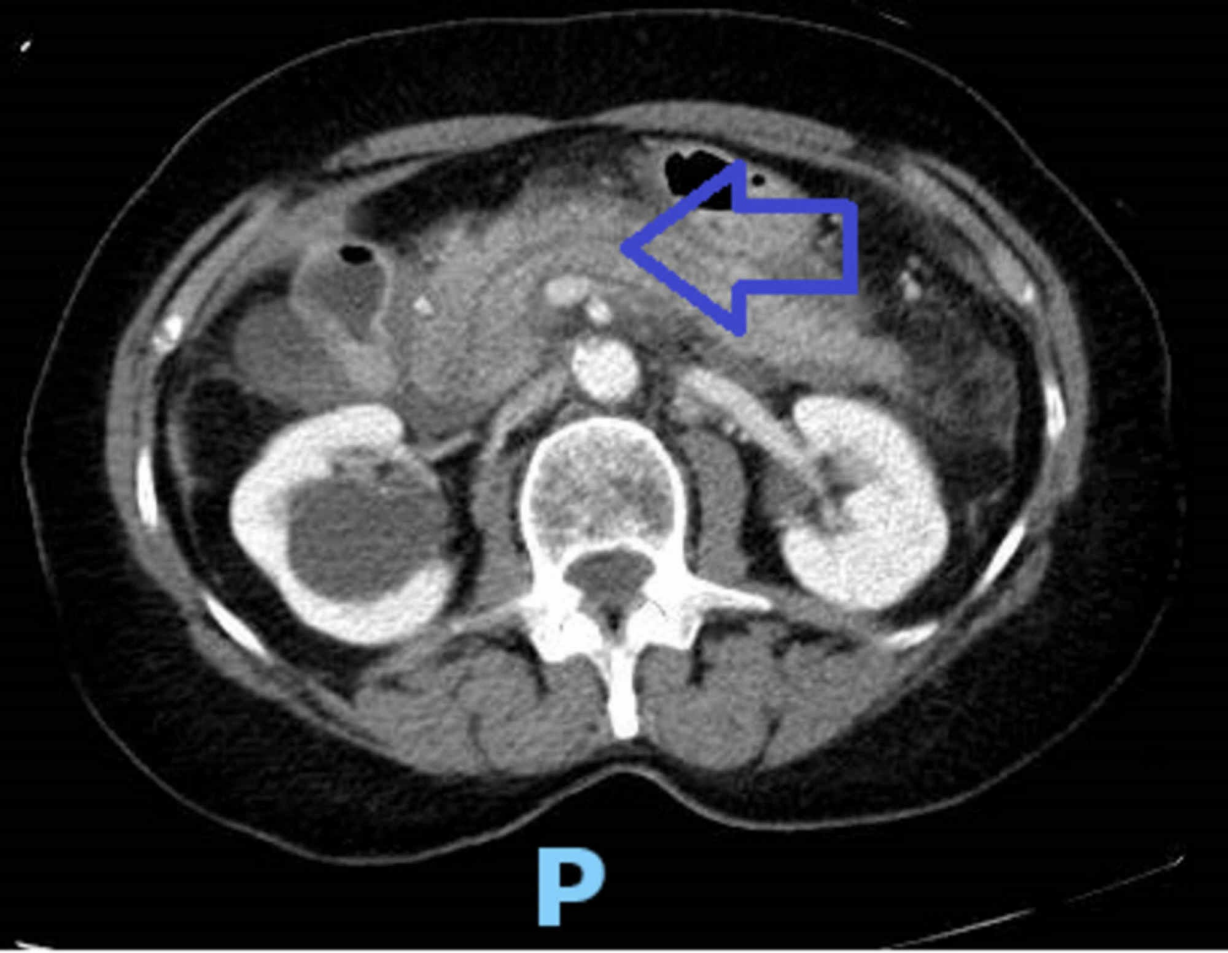
CT abdomen The arrowhead points at the inflamed pancreas.

In this admission, the patient presented with similar complains and the repeat CT abdomen with intravenous (IV) contrast redemonstrated acute pancreatic inflammation with mild peri-pancreatic fluid accumulation without any evidence of necrosis and ductal dilatation (Figure 1). The ultrasound of the abdomen was also repeated, which did not yield any evidence of cholelithiasis and the common bile duct measured 4 mm in size. The patient was evaluated by gastroenterology and was managed symptomatically. MRI the abdomen did not demonstrate intra or extrahepatic biliary ductal pathology, cholelithiasis, or choledocholithiasis. No evidence of abnormal pancreatic ductal pathology was noted. The patient was ultimately taken off losartan and started on calcium channel blocker for hypertension. The patient ultimately improved and was discharged home to do a follow-up with her primary care physician and gastroenterology as needed.

The patient had no evidence of abnormal biliary tract or pancreatic duct pathology on relevant imaging studies. Repeat lipid profile, hepatitis panel and liver function tests were within normal limits. The patient did not have alcohol use disorder, and blood alcohol levels were also negative on both admissions. Losartan was deemed as the causative agent for recurrent pancreatitis, a rare phenomenon to be described in the medical literature.

## Discussion

The aforementioned clinical case describes drug-induced acute pancreatitis (DIAP) with the causative agent being losartan. The literature review has shown that DIAP is rare and has seldom been reported. It is mainly due to a lack of recognition as most of these medications are used frequently. There are no compartmentalizing features and proving the association of the drug with pancreatitis also requires a high degree of suspicion. Drug discontinuation followed by monitoring for the resolution of symptoms of the drug and re-exposure to the same drug causing another episode of acute pancreatitis leads to the diagnosis. Its prevention requires a current knowledge of medications and their possible side-effects. 

Five hundred and fifty drugs are recognized by the World Health Organization (WHO) database to be suspected as a cause of DIAP [1]. Out of these, 525 have been confirmed reported to be associated. It is estimated that DIAP comprises 2% of all cases of acute pancreatitis overall [2]. However, its true incidence is unknown. Causality has been established based on reported cases.

Four categories of medicines have been formulated known to be associated with DIAP. Class I has the list of medications that have at least one case reported as the cause of DIAP. Class IA includes medications that were suspected to be the cause after the most common causes of acute pancreatitis have been excluded. Class IB includes medications that were found to be the cause of DIAP after the rec-challenge of the drug when the common causes could not be ruled out. Class II includes medications that were found to have 75% latency. Class III was not found to have latency and Class IV included medications with very few reported cases and not fitting into other classes [3]. Among inhibitors of the renin-angiotensin system (RAS), reported cases of DIAP are mainly related to the use of captopril, ramipril, enalapril, lisinopril, quinapril, benazepril, losartan, and telmisartan and they are classified as Class IB.

Angiotensin receptor blockers (ARBs) inhibit the binding of molecule angiotensin II at angiotensin type 1 (AT-1) receptors. These receptors are the G-protein coupled receptors with the three other types being AT-2, AT-4, and MAS. All these receptors help regulate cardiovascular, hemodynamic, renal, and endothelial functions in a harmonious way, including cell proliferation, survival, fibroblast proliferation, and inflammation [4]. The net effect is an increase in blood pressure and salt and water retention by vasoconstriction, catecholamine release, increased aldosterone synthesis, vasopressin release, and renal actions [5]. Angiotensin II also exerts its effects by inactivating bradykinin and by the production of prostaglandins. Bradykinin usually causes vasodilation and increased vascular permeability of the post-capillary venules. It leads to extravasation into tissue space, causing angioedema [6-7]. So, ARBs are mainly used to control blood pressure and the common side effects noted are cough, angioedema, hyperkalemia, acute kidney injury, and hypotension.

It is known that AT-I and AT-II receptors are found in pancreatic ducts and acinar cells, which regulate physiological pancreatic functions. Different mechanisms have been proposed through which renin angiotensinogen system inhibitors can induce pancreatitis. The usage of these classes of medications results in decreased bradykinin degradation and increased endothelin 1 affecting the microcirculation of the pancreas and dysregulating the exocrine secretions [8]. Other proposed mechanisms include angioedema of the pancreatic duct and a direct toxic effect on pancreatic cells [9].

Drug-induced acute pancreatitis is always diagnosed after the common causes of acute pancreatitis have been ruled out. Common causes include gallstones, alcoholism, post-trauma, and post-endoscopic retrograde cholangiopancreatography (ERCP). Once these common causes are excluded, drug-induced pancreatitis is diagnosed based on clinical symptoms, elevated levels of serum lipase, amylase, abdominal imaging, and recurrence of symptoms with the reintroduction of the medication [10]. In our clinical case, abdominal imaging studies, including CT abdomen, ultrasound, and MRI, were found to be negative for intra-abdominal pathology. The patient did not have any dyslipidemia, liver function test abnormalities, abnormal hepatitis panel, or elevated blood alcohol levels. The patient was challenged with the reintroduction of losartan and within four weeks of resuming the medication, she developed similar symptoms again, making this highly suspicious for DIAP.

Eland et al., via a prospective case-control study, stated that the risk of DIAP increases with higher loading and maintenance doses [11]. Its incidence is considered highest in the first few months of the onset of therapy. As observed in our case, there was a recurrence within four weeks of restarting losartan therapy. Symptoms resolved on both occasions after losartan discontinuation. A retrospective study conducted by Kuoppala et al. showed a moderately increased risk of DIAP with ACEI [12]. Similarly, Burak et al. described a case of recurrence of pancreatitis on resuming valsartan in a 58-year-old male [13]. He developed the second episode of pancreatitis within six weeks of resuming 160 mg valsartan. It was later discontinued, and the patient then remained symptom-free. In addition, Kanbay et al. shared a case of two episodes of ramipril-induced pancreatitis within 26 months of starting treatment [14]. Upon recognition, it was then replaced by atenolol, and the patient did not have another pancreatitis attack afterward. 

To the best of our knowledge and after thorough literature research, we found three previous case reports of losartan-induced pancreatitis [15-17]. In all these cases, patients were rechallenged with Losartan, which resulted in the recurrence of acute pancreatitis. Pancreatitis being a serious medical condition that can be severe, leading to mortality in about 45% of cases, efforts should be made to further investigate the drug's safety [18]. Given the fact that DIAP is very rare to come across, physicians should have a strong clinical suspicion and insight about these uncommon side effects of commonly prescribed medications including losartan.

## Conclusions

Losartan is a commonly prescribed angiotensinogen receptor blocker used for controlling blood pressure. Drug-induced acute pancreatitis is a rare side-effect associated with this medication and the primary means of its diagnosis is by the exclusion of the common causes of pancreatitis.
